# Diploid genome differentiation conferred by RNA sequencing-based survey of genome-wide polymorphisms throughout homoeologous loci in *Triticum* and *Aegilops*

**DOI:** 10.1186/s12864-020-6664-3

**Published:** 2020-03-20

**Authors:** Sayaka Tanaka, Kentaro Yoshida, Kazuhiro Sato, Shigeo Takumi

**Affiliations:** 1 Graduate School of Agricultural Science, Kobe University, Rokkodai 1-1, Nada-ku Kobe, 657-8501 Japan; 2 Institute of Plant Science and Resources, Okayama University, Chuo 2-20-1, Kurashiki, 710-0046 Japan

**Keywords:** Genome-wide polymorphisms, Genome differentiation, RNA sequencing, Wheat

## Abstract

**Background:**

*Triticum* and *Aegilops* diploid species have morphological and genetic diversity and are crucial genetic resources for wheat breeding. According to the chromosomal pairing-affinity of these species, their genome nomenclatures have been defined. However, evaluations of genome differentiation based on genome-wide nucleotide variations are still limited, especially in the three genomes of the genus *Aegilops*: *Ae. caudata* L. (CC genome), *Ae. comosa* Sibth. et Sm. (MM genome), and *Ae. uniaristata* Vis. (NN genome). To reveal the genome differentiation of these diploid species, we first performed RNA-seq-based polymorphic analyses for C, M, and N genomes, and then expanded the analysis to include the 12 diploid species of *Triticum* and *Aegilops*.

**Results:**

Genetic divergence of the exon regions throughout the entire chromosomes in the M and N genomes was larger than that between A- and A^m^-genomes. *Ae. caudata* had the second highest genetic diversity following *Ae. speltoides*, the putative B genome donor of common wheat. In the phylogenetic trees derived from the nuclear and chloroplast genome-wide polymorphism data, the C, D, M, N, U, and S genome species were connected with short internal branches, suggesting that these diploid species emerged during a relatively short period in the evolutionary process. The highly consistent nuclear and chloroplast phylogenetic topologies indicated that nuclear and chloroplast genomes of the diploid *Triticum* and *Aegilops* species coevolved after their diversification into each genome, accounting for most of the genome differentiation among the diploid species.

**Conclusions:**

RNA-sequencing-based analyses successfully evaluated genome differentiation among the diploid *Triticum* and *Aegilops* species and supported the chromosome-pairing-based genome nomenclature system, except for the position of *Ae. speltoides*. Phylogenomic and epigenetic analyses of intergenic and centromeric regions could be essential for clarifying the mechanisms behind this inconsistency.

## Background

Crop domestication first occurred more than 10,000 years before the present. Since the early domestication process, ancient and modern breeders have utilized related wild species as genetic resources for crop improvement [1]. Recent and future climate change requires more efficient use of the useful genes in wild relatives [2, 3]. Elucidating the precise phylogenetic relationships among crops and their wild relatives will provide basic information for the use of agriculturally important genes found in the wild.

Genera *Triticum* and *Aegilops* include diverse diploid and allopolyploid species. The allopolyploid species are allotetraploids and allohexaploids, which were established through interspecific crossings between close and distinct relatives followed by chromosome doubling. In addition to allopolyploidization, nuclear differentiation at the diploid level drives speciation in this plant group. Genome differentiation was initially defined and updated based on the bivalent formation in meiotic cells of interspecific hybrids among related species in *Triticum* and *Aegilops* [4, 5]. The homoeologous chromosomes of the diploid genomes are distinguished by in situ hybridization patterns of highly repetitive sequences and C-banding patterns, indicating that genome differentiation of diploid wheat and its relatives manifests at least partly in the distribution of heterochromatin and the accumulation of highly repetitive sequences [6]. Certain repetitive sequences such as retrotransposons rapidly and dramatically increase in their copy numbers in evolutionary-specific lineages [7–9], implying that repetitive sequence-based approaches would not necessarily reflect genetic relationships among related species. The use of genome-wide exon sequences, therefore, should be considered for clarifying the evolutionary relationships among related genomes.

Comprehensive studies on organellar genome diversity among *Triticum* and *Aegilops* using alloplasmic lines of common wheat have revealed diverse effects of differentiated chloroplast and mitochondrial genomes on various phenotypic and physiological traits [10–13]. The phylogenetic tree of organellar genomes is based on the maternal parents of *Triticum* and *Aegilops* allopolyploids and phylogenetic relationships among the organellar genomes of diploid species. Mitochondrial genomes have diverged in parallel with the chloroplast genomes of *Triticum* and *Aegilops* [12, 13]. Organellar DNA variations are significantly correlated with phenotypes in alloplasmic wheat lines [12]. Studies based on chloroplast nucleotide sequences have also clarified the phylogenetic relationships among chloroplast genomes in the tribe Triticeae, including the diploid *Triticum* and *Aegilops* species [14, 15]. According to these previous reports, the phylogenetic relationship of the organellar genomes among *Triticum* and *Aegilops* is inconsistent with the one based on chromosome-pairing affinity. The position of *Aegilops speltoides* Tausch, an organellar genome donor of tetraploid and hexaploid wheat species, is especially discordant between the chromosome-pairing-based and organellar genome-based methods.

RNA sequencing (RNA-seq) has been a useful approach to survey genome-wide polymorphisms, including single-nucleotide polymorphisms (SNPs) and insertions/deletions (indels), in several wheat diploid relatives [16–23]. RNA-seq-derived polymorphism information is readily available to develop PCR-based markers such as cleaved amplified polymorphic sequences (CAPS) in target chromosomal regions. In this study, we conducted RNA-seq analyses for three diploid *Aegilops* species, namely *Ae. caudata* L. (syn. *Ae. markgrafii* Hammer, CC genome), *Ae. uniaristata* Vis. (NN genome), and *Ae. comosa* Sibth. et Sm. (MM genome). The three species are useful genetic resources for introgression of disease resistance into common wheat [24, 25]. *Aegilops caudata* accessions are distributed from Greece to the northern part of Iraq [26]. *Ae. uniaristata* and *Ae. comosa* belong to the section Comopyrum, and have limited distribution in northwestern Turkey and from northwestern Turkey to Greece, respectively [27]. Comopyrum species are utilized for identifying novel alleles of glutenin subunit genes [28, 29]. Despite their usefulness as genetic resources, little genome information has been accumulated from these three *Aegilops* species.

The research objectives of the present study were (1) to survey RNA-seq-based polymorphisms through all chromosomes in the C, M, and N genome diploid species, (2) to convert the polymorphisms into genome-specific PCR-based markers, and (3) to clarify the phylogenetic relationships among the diploid *Triticum* and *Aegilops* species using exon-derived genome-wide polymorphism data.

## Results

### Genome-wide genetic variations in three diploid *Aegilops* species

**Fig. 1:**
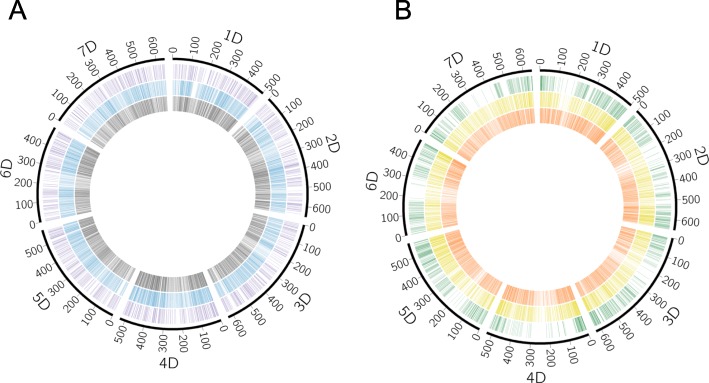
Distribution of polymorphic sites and fixed SNPs within/between *Aegilops caudata* (CC genome), *Ae. comosa* (MM genome), and *Ae. uniaristata* (NN genome). **a** The CIRCOS plot visualizes polymorphic sites within species. Violet, blue, and black lines indicate polymorphic sites within *Ae. uniaristata*, *Ae. Comosa*, and *Ae. caudata*, respectively. **b** Green, yellow, and orange lines indicate fixed SNPs between *Ae. comosa* and *Ae. uniaristata*, between *Ae. caudata* and *Ae. comosa*, and between *Ae. caudata* and *Ae. uniaristata*, respectively

To clarify the nucleotide variations in *Ae. caudate* (CC genome), *Ae. uniaristata* (NN genome), and *Ae. comosa* (MM genome), RNA-seq for a total of 15 accessions of these species was performed (Additional file 1: Fig. S1 and Table S1), generating 4,530,173 to 6,296,846 paired reads for each accession. After filtering out low-quality reads, 3,007,539 to 5,040,664 read pairs were obtained for the subsequent analyses (Additional file 1: Table S2). Of the filtered reads, 66.86 to 97.24% were aligned to *Ae. tauschii* genome sequences (Additional file 1: Table S3). Alignment rate variations were detected between the accessions of each species, and the alignment rate was not dependent on species. SNP and indel calling based on the short read alignments identified 13,401 to 135,902 SNPs and 177 to 1646 indels between *Ae. caudata* and *Ae. tauschii*, 14,880 to 86,171 SNPs and 220 to 1528 indels between *Ae. comosa* and *Ae. tauschii*, and 20,901 to 184,593 SNPs and 278 to 2273 indels between *Ae. uniaristata* and *Ae. tauschii* (Additional file 1: Table S3). These SNPs and indels covered all the chromosomes of *Ae. tauschii* (Additional file 1: Fig. S2). Of these SNPs, 83,018, 61,704, and 106,652 sites were polymorphic in *Ae. caudate*, *Ae. comosa*, and *Ae. uniaristata*, respectively (Additional file 1: Table S4). The distributions of the polymorphic sites over the chromosomes were not strikingly different among the three species (Fig. 1a and Additional file 1: Table S4).

### Development of M and N genome-specific markers and their utility

To develop M and N genome-specific makers, we identified 13,600 fixed SNPs between *Ae. comosa* (MM genome) and *Ae. uniaristata* (NN genome) that can discriminate M and N genomes. A fixed SNP site is monomorphic within a species, while it has different nucleotides between species. These fixed SNPs between *Ae. comosa* and *Ae. uniaristata* covered all the chromosomes (Fig. 1b). Each chromosome had 1729 to 2249 fixed SNPs (Additional file 1: Table S5). When compared to the number of fixed SNPs between *Ae. comosa* and *Ae. caudata* and between *Ae. uniaristata* and *Ae. caudata*, the number of fixed SNPs between *Ae. comosa* and *Ae. uniaristata* was small. This result is consistent with the taxonomic classification: these two species belong to the same section Comopyrum. Three CAPS markers were designed based on these fixed SNPs (Additional file S1: Fig. S3 and Table S6). These CAPS markers successfully discriminated N and M genomes.

### Phylogenetic relationships among diploid *Triticum* and *Aegilops* species based on SNPs in the coding regions of nuclear genomes

**Fig. 2:**
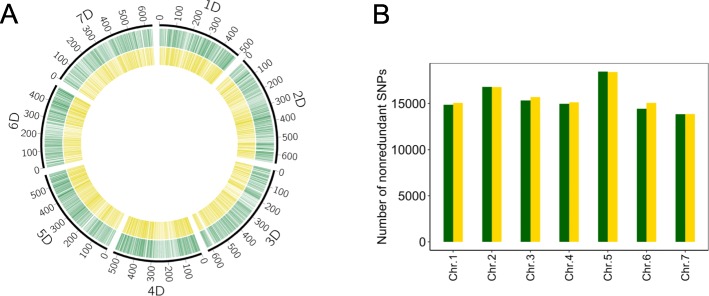
Distribution of non-redundant SNPs over the chromosomes of nuclear genomes. Distributions of non-redundant SNPs with/without outgroup species are visualized by a CIRCOS plot (**a**). Green and yellow lines represent positions of non-redundant SNPs with and without outgroups species over the chromosomes, respectively. The number of non-redundant SNPs for each chromosome is shown as a barplot (**b**). Green and yellow bars indicate non-redundant SNPs with and without outgroup species, respectively

To reveal the phylogenetic relationships of diploid *Triticum* and *Aegilops* species, we utilized the previously published RNA-seq data of *Ae. tauschii* (DD genome) [19], *Ae. umbellulata* (UU genome) [20], einkorn wheat (AA and A^m^A^m^ genomes) [23], and Stiopsis species (SS genome) [21], combining it with our current data from *Ae. caudat*a (CC genome), *Ae. comosa* (MM genome), and *Ae. uniaristata* (NN genome) (Additional file 1: Table S7). The qualified 300 bp paired-end short reads of all the species were aligned to the *Ae. tauschii* genome sequences (Additional file 1: Table S8), generating a set of 109,980 non-redundant SNPs (Additional file 1: Table S9). Considering that the non-redundant SNPs were distributed over all the chromosomes (Fig. 2), SNPs could be regarded as representative SNPs that adequately reflect the nuclear genome evolution of the diploid *Aegilops*/*Triticum* species. Another set of 108,618 non-redundant SNPs for the diploid *Aegilops*/*Triticum* species, including *Hordeum vulgare* as an outgroup species, was prepared for the phylogenetic analyses (Fig. 2 and Additional file 1: Table S9). Due to the lower alignment rate of *H. vulgare* to RNA-seq reads of the *Ae. tauschii* reference genome (Additional file 1: Table S8), the number of non-redundant SNPs within the diploid *Triticum* and *Aegilops* species was reduced when *H. vulgare* was included (Additional file 1: Table S9).

**Fig. 3:**
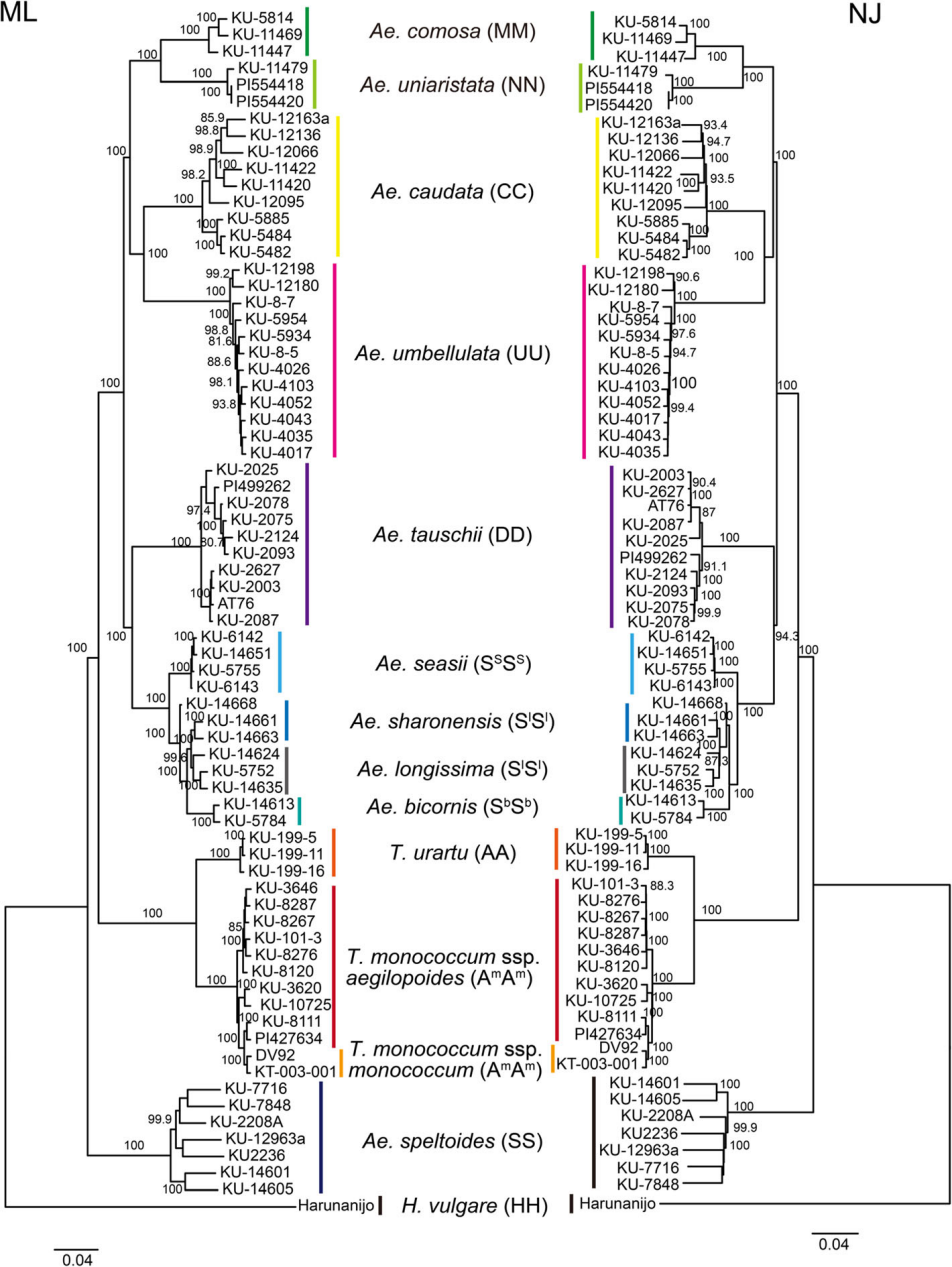
Phylogenetic relationship among diploid *Triticum* and *Aegilops* species. A maximum-likelihood tree and a neighbor-joining tree are shown. The trees were constructed based on 108,618 non-redundant SNPs in the nuclear genome. The number next to each branch indicates bootstrap probability based on 1000 replications

Phylogenetic trees of the diploid *Triticum* and *Aegilops* species were constructed using neighbor-joining (NJ) and maximum likelihood (ML) methods (Fig. 3). All the phylogenetic trees with/without outgroup species *H. vulgare* showed the same topology, which was consistent with the topology of the previously reported phylogenetic trees based on RNA-seq [22]. The diploid species having the same genome were classified into the same clades with 100% bootstrap probability, except for Sitopsis species. Section Sitopsis was separated into two clades that correspond to the subsections Emaginata and Truncata [21, 22]. Subsection Emaginata was more closely related to D-genome species. As reported by Glémin et al. 2019 [22], *Triticum* and *Aegilops* species are classified into three large clades: einkorn wheat (A and A^m^ genomes), Truncata (S genomes), and other species (C, D, M, N, U, and S genomes that were further classified into S^s^S^s^, S^l^S^l^, and S^b^S^b^). As expected**,** M and N genome species belonging to the section Comopyrum had the closest relationship. C genome species were more closely related to U genome species than to M and N genome species. The branch length between M and N genome species was longer than that between A and A^m^ genome species, and was slightly smaller than that between C and U genome species.

**Fig. 4:**
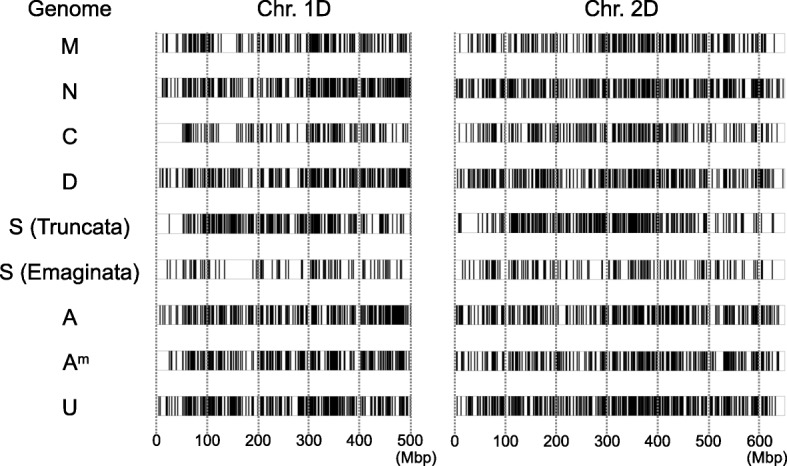
Distribution of unique SNPs that discriminated between genomes over each chromosome. The unique SNPs for each genome were mapped to the chromosomes of *Ae. tauschii*. Black bars indicate SNP positions. The figure shows the distribution of the unique SNPs on chromosomes 1D and 2D. The results for other chromosomes are shown in Additional file S1: Fig. S4

Since the phylogenetic tree confirmed the genome differentiation between the diploid species, we investigated the distribution of unique nucleotide substitutions over the chromosomes that discriminated between each of the genomes (Fig. 4 and Additional file S1: Fig. S4). When non-redundant SNPs were monomorphic within a species and distinct from the other diploid species of *Aegilops* and *Triticum*, they were regarded as unique nucleotide substitutions. In this analysis, the S genome species of the section Emaginata were assembled into one group. In every genome, unique nucleotide substitutions covered all chromosomes with some differences in their density.

### Nucleotide polymorphisms within each nuclear genome

**Fig. 5:**
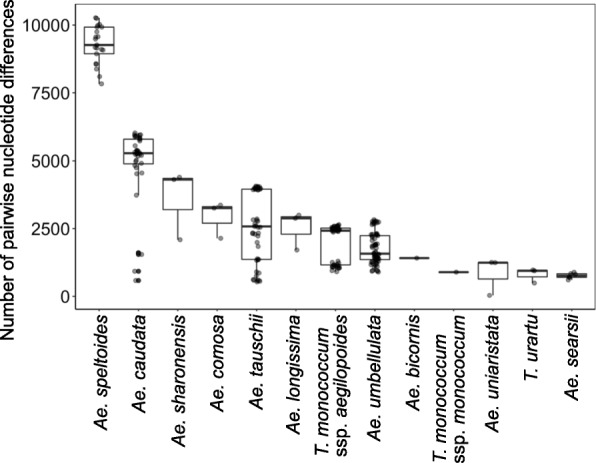
Distinct genetic diversity among diploid *Triticum* and *Aegilops* species. A boxplot with jitter points representing the number of nucleotide difference between individual accessions within species is shown. Each translucent grey point indicates one pairwise comparison between two accessions. Darker points indicate overlaps of points. The median of each species in the boxplot clarifies distinct genetic diversity between species and jitter points disclose discontinuities in nucleotide differences between accessions within species

To evaluate the level of nucleotide polymorphisms for diploid *Triticum* and *Aegilops* species, we used the number of pairwise nucleotide differences between accessions within species as an indicator of genetic diversity (dissimilarity), which was calculated based on the set of non-redundant SNPs excluding *H. vulgare*. The usage of non-redundant SNPs without missing values enables us to compare genetic diversity among species on an equal basis. Genetic diversity was quite distinct among the diploid *Triticum* and *Aegilops* species (Fig. 5). Following *Ae. speltoides*, *Ae. caudata* had the second highest genetic diversity among the diploid *Triticum* and *Aegilops* species. In *Ae. caudata*, *Ae. tauschii*, and *T. monococcum* ssp. *aegilopoides* (Link) Thell. (syn. *T. boeoticum* Boiss), the number of pairwise nucleotide differences depended on the pairs of accessions, implying the existence of genetically divergent groups within their species. This observation is consistent with previous reports of *Ae. tauschii* and *T. monococcum* ssp. *aegilopoides* indicating that these two species contain more than two divergent groups [19, 23, 30]. *T. urartu*, *T. monococcum* ssp. *monococcum*, and *Ae. searsii* showed lower genetic diversity than the other diploid *Triticum* and *Aegilops* species.

### Phylogenetic relationships of the organelle genomes of diploid *Triticum* and *Aegilops* species

**Fig. 6:**
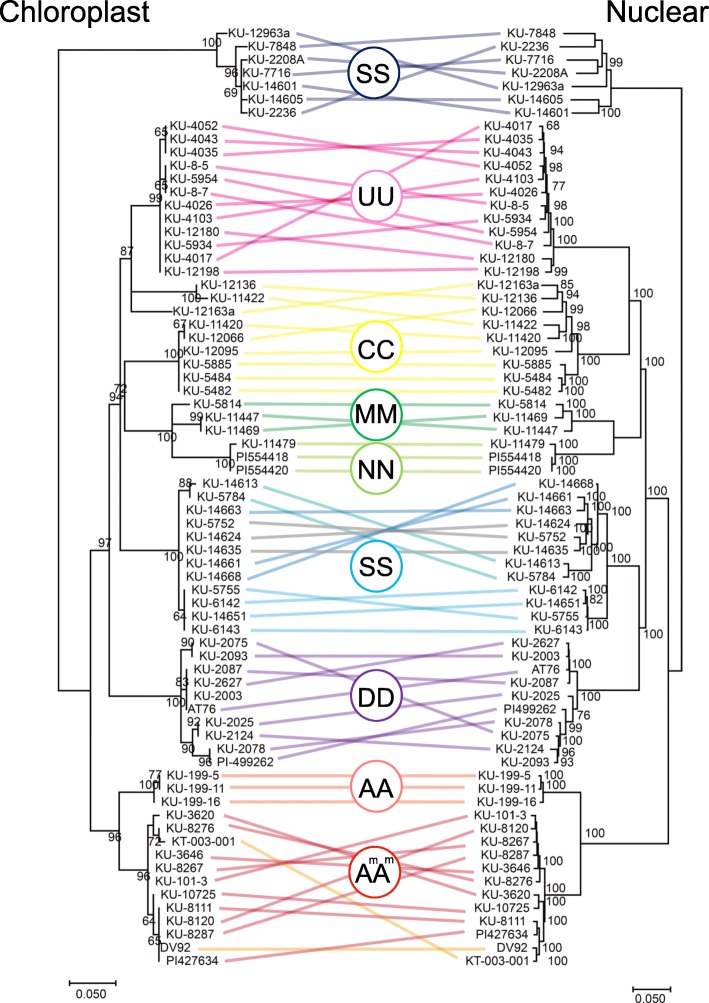
Genome differentiation of chloroplasts and nuclei of diploid *Triticum* and *Aegilops* species. Maximum likelihood phylogenetic trees based on 234 non-redundant SNPs of chloroplasts and nuclei are shown. The same accessions in the trees are connected with colored lines. Different colors are used for each species. Letters in the colored circles represent genomes. Bootstrap probabilities based on 1000 replications are shown next to the branches

RNA-seq short reads of the diploid *Triticum* and *Aegilops* species were aligned to the chloroplast genome of *Ae. tauschii*. The alignment rate of short reads was dependent on the accessions (Additional file 1: Table S3 and Table S8), and the alignment rate for some accessions was over 30%. This high percentage could be due to a large amount of chloroplast RNA contained in the sampled leaves from these accessions and/or could result from misalignment of RNA-seq short reads that should be mapped to the nuclear genome. After detecting SNPs for each accession and combining them, we obtained 234 non-redundant SNPs in the chloroplast genome. In order to address organelle genome evolution, a phylogenetic tree was constructed based on these non-redundant SNPs using the ML method (Fig. 6). The topology of the phylogenetic tree was highly consistent with that based on SNPs of the nuclear genome, but the following minor differences existed in the topology. In the chloroplast genome, after separation from the einkorn wheat (AA and A^m^A^m^ genomes) and *Ae. speltoides* (SS genome), *Ae. tauschii* (DD genome) first diverged from the other *Aegilops* species. Also, *Ae. caudata* (CC genome) showed a non-monophyletic pattern. Three accessions of *Ae. caudata* were more closely related to *Ae. umbellulata* (UU genome), while the other accessions of *Ae. caudata* were close to *Ae. comosa* (MM genome) and *Ae. uniaristata* (NN genome). In the nuclear trees, S genome species for subsection Emaginata and D, C, M, N, and U genome species formed a monophyletic clade, indicating that they diverged from one common ancestor, and *Ae. caudata* was a monophyletic group.

## Discussion

### Clear differentiation between *Ae. comosa* and *Ae. uniaristata* despite their phenotypic similarity

Our RNA-seq-based phylogenetic analyses using SNPs in nuclear and chloroplast genomes showed that *Ae. uniaristata* and *Ae. comosa*, belonging to the section Comopyrum, were the most closely related species among the diploid *Triticum* and *Aegilops* species. Both species belonged to a monophyletic clade, suggesting that they originated from one common ancestor. This observation is consistent with the nuclear and chloroplast phylogenetic relationships of published studies that have used different sets of accessions and the different methods for detecting nucleotide variations [15, 22].

Our study indicates high genetic divergence between *Ae. uniaristata* and *Ae. comosa,* which was higher than that between A and A^m^ genomes (Fig. 3), even though the morphologies of *Ae. uniaristata* and *Ae. comosa* are similar. Unique nucleotide substitutions that discriminate them from other genomes were distributed over the chromosomes in both species (Fig. 4 and Additional file S1: Fig. S4). Considering that coding regions are generally more conservative than intergenic regions, which are mostly composed of repetitive sequences and transposable elements, the intergenic regions are expected to have higher genetic divergence. In fact, there are distinct in situ hybridization patterns of highly repetitive sequences and C-banding patterns between M and N genomes [6]. Nucleotide differences between both species may thus cause non-preferential chromosome pairing between M and N genomes [31]. Whole genome sequence comparisons, including intergenic regions, will be necessary for understanding the relationship between genome differentiation and chromosome-pairing affinity.

### Genome differentiation in nuclear and chloroplast genomes in diploid *Triticum* and *Aegilops* species

The observed short internal branches in the phylogenetic trees of nuclear and chloroplast genomes suggest that *Triticum* and *Aegilops* species emerged during a relatively short period in the past and then the nuclear and chloroplast genomes each diverged (Fig. 6). For the nuclear genome, first, the S genome of the section Truncata was separated from the other genomes, and then the A and A^m^ genomes of einkorn species were separated from a common ancestor of S, C, D, M, N, and U genomes (Figs. 3and 6). S, D, M, N, and U genomes form a monophyletic clade. Their common ancestor diverged into two groups: one is composed of U, C, M, and N genomes, and the other is of S and D genomes. This observation is consistent with a previously proposed scenario of the evolutionary history of *Aegilops/Triticum* species [22]. In contrast, for the chloroplast genome, after separating from A and A^m^ genomes, the D genome diverged from the C, D, M, N, and S genomes. The C genome species exhibited a polyphyletic relationship. Considering that these minor inconsistencies between the nuclear and chloroplast genomes were observed only in the short internal branches of the phylogenetic tree (Fig. 6), genome differentiation of the chloroplast might have occurred independently of that of the nucleus during the short emergence period of S, D, M, N, and U genomes. On the other hand, considering the close consistency of the phylogenetic relationships between the nuclear and chloroplast genomes, both genomes could have coevolved after diversification of each genome.

The phylogenetic relationship of the organellar genomes of *Triticum* and *Aegilops* is inconsistent with their chromosome-pairing affinity [12–15]. In particular, the chromosome-pairing-based and organellar genome-based relationships conflict at the position of *Ae. speltoides*. The current and previous phylogenetic analyses using RNA-seq based SNPs indicate that *Ae. speltoides* (SS genome) belonging to the section Truncata is separate from the other SS genome species belonging to the section Emaginata [21, 22]. There are apparent discrepancies in the relationships of S genomes between the exon sequences of their nuclear genomes and chromosome-pairing affinity. Intergenic regions, including transposable elements and centromeric regions, occupy most of the genomes. Meiotic associations of centromeres precede chromosome arm pairing. After initial associations of nonhomologous centromeres, homologous centromere pairing is considered to be established by assessments of DNA sequence homology, although the mechanisms behind centromere paring are not fully understood [32]. The study of fission yeast implies that noncoding RNA is involved in homologous chromosome pairing [33]. Supportive results of heterochromatic-mediated paring have been found in *Caenorhabditis elegans*, *Drosophila*, and *Arabidopsis* [32]. Intergenic regions and epigenetic mechanisms potentially contribute to the chromosome-pairing affinity among *Triticum* and *Aegilops* species. Phylogenomic and epigenetic analyses of the intergenic regions may provide clues for solving the contradiction between the nuclear and chloroplast genomes and the chromosome-pairing affinity.

Some SNPs from chloroplast genomes are potentially derived from mutations by RNA editing [34]. These SNPs, however, should affect only the length of the external branches in the phylogenetic tree. Informative SNPs for the tree construction could be caused by nucleotide substitutions in the evolutionary history of *Triticum*/*Aegilops* species rather than mutations via RNA editing, because RNA editing occurs in mRNA from each chloroplast in each individual species. Nucleotide sequences of chloroplast genomes from RNA-seq represent average nucleotide sequences of mRNA from the chloroplast genome of an individual. Most SNPs produced via RNA editing can be detected as heterozygous SNPs. Our analysis used only homozygous SNPs and enabled us to reduce the effects of RNA editing on the topology of the phylogenetic trees.

### Nucleotide polymorphisms in diploid *Triticum* and *Aegilops* species

*Ae. speltoides* had the highest nucleotide diversity among the tested diploid species. Only *Ae. speltoides* is an outcrossing species, while the others in our study are selfing species. The mating system of outcrossing increases effective population size and recombination events, both of which positively affect genome-wide genetic diversity [35]. Although RNA-seq can detect only SNPs in expressed genes, the detected SNPs covered all the chromosomes. The number of nucleotide differences between individuals could be regarded as a representative value of genome-wide genetic diversity.

Although it is possible to underestimate nucleotide diversity due to the small number of analyzed accessions in some species, our study revealed varying levels of genetic diversity among *T. monococcum ssp. aegilopoides*, *Ae. caudata*, *Ae. umbellulata*, and *Ae. tauschii*, although they are all selfing species (Fig. 5). Their effective population sizes, population structures, and demographic histories such as speed and time of colonization of their modern habitats could be the factors that generated distinct genetic diversity. After *Ae. speltoides*, which is an outcrossing species, *Ae. caudata* had the second highest nucleotide diversity. In the phylogenetic tree of the nuclear genome, Turkish and Iraqi accessions (KU-5482, KU-5484, KU-5885) and Greek accessions of *Ae. caudata* were genetically divergent (Fig. 3). Greek accessions had long external branches. If individuals of *Ae. caudata* are regarded as a representative of a local population, genetic differences between the Greek accessions could reflect isolation between local populations.

Genetic diversity in *Ae. tauschii* was higher than that in *T. monococcum* ssp. *aegilopoides* and *Ae. umbellulata*. *Ae. tauschii* has the broadest range of habitats from the Middle East to China. Three genetically divergent sublineages, TauL1, TauL2, and TauL3, are detected in *Ae. tauschii* [30]. Genetic differences between the sublineages explain the higher genetic diversity in *Ae. tauschii* [19]. These sublineages have contrasting geographical distributions. TauL2 is mainly distributed in Transcaucasia and the Middle East. The TauL1 sublineage can be divided into TauL1a and TauL1b. The habitat of TauL1a is endemic to the Transcaucasus and the Middle East, while the habitat of TauL1b expands to East Asia. The phenological change to early flowering and the gain of NaCl-induced-stress tolerance in TauL1b have contributed to its colonization of semi-arid Central Asian habitats [36, 37]. The higher genetic diversity could be linked to its successful colonization of diverse habitats, which may have accelerated genetic differentiation between the sublineages due to the limited gene flow between its remote habitats.

The observed pattern of *T. monococcum* ssp. *aegilopoides* was caused by nucleotide divergence between two groups, one of which includes the ancestor of the diploid cultivar *T. monococcum* ssp. *monococcum* [23]. On the other hand, *Ae. umbellulata* had lower genetic diversity and a star-like phylogeny with short external branches, suggesting recent expansion into modern habitats [20]. Polyphyly of *Ae. caudata* has been reported in a previous study based on four regions of the chloroplast genome [14]. In this study, the chloroplast genomes of *Ae. caudata* also showed a polyphyletic pattern. One of the groups was closer to *Ae. umbellulata*, while the others were closer to *Ae. comosa* and *Ae. uniaristata*.

## Conclusions

The RNA-seq approach uncovered genetic divergence of the exon regions through all of the chromosomes of C, M, and N genome diploid species, allowing us to construct novel CAPS makers that discriminated between M and N genomes. Phylogenetic trees based on the exon-derived genome-wide polymorphism data overall supported the differentiation of nuclear and chloroplast genomes of diploid *Triticum* and *Aegilops* species. Chromosomal pairing-affinity between S genomes, however, was contradicted by their genetic differentiation of exon regions. In order to resolve this inconsistency, phylogenomic and epigenetic analyses of intergenic regions and centromeric regions are needed in future studies.

## Methods

### Plant materials

Seeds of nine accessions of *Ae. caudata*, three accessions of *Ae. uniaristata*, and three accessions of *Ae. comosa* were supplied by the National BioResource Project (NBRP)-Wheat, Japan (https://www.nbrp.jp) for KU-numbered accessions and by the National Small Grains Research Facility, USDA-ARS, USA for PI-numbered accessions (Additional file 1: Table S1). These accessions were collected in Greece, Iraq, and Turkey.

### RNA sequencing

Total RNA was extracted using Sepasol-RNA I Super G (Nacalai Tesque, Kyoto, Japan) from the mature leaves of 2-month-old plants in the field of Kobe University, Kobe, Japan. After DNase I treatment, libraries for 300-bp paired-end RNA-seq were constructed using 6 to 10 μg of total RNA using the TruSeq RNA Library Preparation Kit v2 (Illumina, San Diego, CA, USA) according to a previously reported protocol [38]. Sequencing was conducted on an Illumina MiSeq sequencer. The obtained reads were deposited to the DDBJ Sequence Read Archive under accession number DRA009411. To evaluate genome differentiation between the diploid *Triticum* and *Aegilops* species, we also used published RNA-seq reads that have been generated using the same protocol and sequencing platform: 10 accessions of *Ae. tauschii* [19], 12 accessions of *Ae. umbellulata* [20], 19 accessions of the section Sitopsis, 15 accessions of the einkorn wheat [23], and one accession of *H. vulgare* [38] (Additional file 1: Table S7). These RNA-seq reads were obtained from the DDBJ Sequence Read Archives.

### Quality control, alignment of RNA-seq reads, and molecular evolutionary analyses

Quality control was conducted according to Miki et al. [21]. Adapter sequences and low-quality bases with an average quality score per 4 bp of < 30 were removed, and reads of fewer than 50 bp were filtered out using Trimmomatic software, version 0.33 [39]. The qualified paired reads were aligned to the reference genome sequence of *Ae. tauschii* [40], and the chloroplast genomes (NCBI reference sequence: NC_022133.1) were aligned using HISAT software, version 2.1.0 [41]. The same procedure as described by Nishijima et al. (2016) [19] was used to detect SNPs and indels. SNPs and indels were called using Coval [42] under the following criteria: the depth of read coverage was ≥10, and > 95% of the aligned reads designated different nucleotides from the referenced ones. We chose positions of SNPs at which no ambiguous nucleotides were detected and the read depth was ≥10, generating a set of nonredundant SNPs for nuclear, chloroplast, and mitochondrial genomes of the tested species. Based on nonredundant SNPs, fixed SNPs that distinguished C, M, and N genomes were detected. CIRCOS, version 0.33 [43] was used for visualization of SNP/indel distributions over the chromosomes of *Ae. tauschii*. Phylogenetic trees were constructed based on the set of nonredundant SNPs. MEGA, version 10 [44] was used for the construction of maximum likelihood (ML) trees and neighbor-joining trees (NJ).

### Marker development

CAPS markers were designed based on the fixed SNPs between M- and N- genomes. Primer sets were generated with Primer3plus software [45] (Additional data 1: Table S6). The candidate SNPs were converted to CAPS markers. PCR amplification, digestion of the PCR products, and visualization of their digested fragments were conducted as described in our previous study [46].

## Supplementary information

**Additional file 1: Table S1.** Accessions of C, M, and N genome species used for RNA-seq analyses. **Table S2.** Quality control of RNA-seq reads of C, M, N-genome diploid wheat accessions. **Table S3.** Alignment rate and SNPs and indels for C, M, and N genome species. **Table S4.** Polymorphic sites in *Ae. caudata* (CC genome), *Ae. comosa* (MM genome), and *Ae. uniaristata* (NN genome). **Table S5.** Fixed SNPs between *Ae. caudata* (CC genome), *Ae. comosa* (MM genome), and *Ae. uniaristata* (NN genome). **Table S6** List of CAPS markers to distinguish M and N genomes. **Table S7** Plant materials used for phylogenetic and polymorphic analyses. **Table S8** Alignment rate and SNPs and indels for diploid *Triticum* and *Aegilops* species used for phylogenetic and polymorphic analyses. **Table S9** Sets of non-redundant SNPs in diploid *Triticum* and *Aegilops* species estimated using RNA-seq. **Figure S1** Geographic distribution of diploid *Triticum* and *Aegilops* species that were analyzed in this study. **Figure S2** Distribution of SNPs and indels of *Ae. caudata*, *Ae. comosa*, and *Ae. uniaristata*. In the CIRCOS visualizations, green, yellow, and orange indicate *Ae. uniaristata*, *Ae. comosa*, and *Ae. caudata*, respectively. SNPs (A) and indels (B) for *Ae. uniaristata* KU-11479, PI554418, and PI554420; *Ae. comosa* KU-5814, KU-11447, and KU-11469; and *Ae. caudata* KU-5482, KU-5484, KU-5885, KU-11420, KU-11422, KU-12066, KU-12095, KU-12136, and KU-12136a are shown from the outer to the inner circles. **Figure S3** CAPS markers that discriminate M or N genomes from A and B genomes. **Figure S4** Distribution of unique SNPs that discriminate other genomes over each chromosome. The unique SNPs for each genome were mapped to the chromosomes of *Ae. tauschii*. Black bars indicate SNP positions. The figure shows the distribution of the unique SNPs on the chromosomes 3D, 4D, 5D, 6D, and 7D.

## Data Availability

All data generated or analyzed during this study are included in this published article in Additional file 1 and DDBJ Sequence Read Archive under accession number DRA009411. The sets of unique SNPs for each species that were used for Fig. 4 and Additional file S1: Fig. S4 are available at https://github.com/PlantGeneticsKobeU/Diploid-Aegilops-species-unique-SNPs.

## Abbreviations

RNA-seq: RNA-sequencing
SNPs: Single nucleotide polymorphisms
CAPS: Cleaved amplified polymorphic sequences
PCR: Polymerase chain reaction
ML: Maximum likelihood
NJ: Neighbor-joining
