# Supplementary material for: Diploid genome differentiation conferred by RNA sequencing-based survey of genome-wide polymorphisms throughout homoeologous loci in Triticum and Aegilops
Source: BMC Genomics. 2020 Mar 20;21:246. doi: 10.1186/s12864-020-6664-3 (PMC7083043; doi:10.1186/s12864-020-6664-3)
Supplement: Supplementary file 1 — Additional file 1: Table S1. Accessions of C, M, and N genome species used for RNA-seq analyses. Table S2. Quality control of RNA-seq reads of C, M, N-genome diploid wheat accessions. Table S3. Alignment rate and SNPs and indels for C, M, and N genome species. Table S4. Polymorphic sites in Ae. caudata (CC genome), Ae. comosa (MM genome), and Ae. uniaristata (NN genome). Table S5. Fixed SNPs between Ae. caudata (CC genome), Ae. comosa (MM genome), and Ae. uniaristata (NN genome). Table S6 List of CAPS markers to distinguish M and N genomes. Table S7 Plant materials used for phylogenetic and polymorphic analyses. Table S8 Alignment rate and SNPs and indels for diploid Triticum and Aegilops species used for phylogenetic and polymorphic analyses. Table S9 Sets of non-redundant SNPs in diploid Triticum and Aegilops species estimated using RNA-seq. Figure S1 Geographic distribution of diploid Triticum and Aegilops species that were analyzed in this study. Figure S2 Distribution of SNPs and indels of Ae. caudata, Ae. comosa, and Ae. uniaristata. In the CIRCOS visualizations, green, yellow, and orange indicate Ae. uniaristata, Ae. comosa, and Ae. caudata, respectively. SNPs (A) and indels (B) for Ae. uniaristata KU-11479, PI554418, and PI554420; Ae. comosa KU-5814, KU-11447, and KU-11469; and Ae. caudata KU-5482, KU-5484, KU-5885, KU-11420, KU-11422, KU-12066, KU-12095, KU-12136, and KU-12136a are shown from the outer to the inner circles. Figure S3 CAPS markers that discriminate M or N genomes from A and B genomes. Figure S4 Distribution of unique SNPs that discriminate other genomes over each chromosome. The unique SNPs for each genome were mapped to the chromosomes of Ae. tauschii. Black bars indicate SNP positions. The figure shows the distribution of the unique SNPs on the chromosomes 3D, 4D, 5D, 6D, and 7D. [file 12864_2020_6664_MOESM1_ESM.pdf]

Table S1 Accessions of C, M and N genome species used for RNA-seq analyses

Species	Strain name	Country	Locality	Year	RNA-seq read data
Aegilops caudata L.	KU-5482	Iraq	13.4 km W from Amadiyah to Bamarni	1971	In this study
Ae. caudata L.	KU-5484	Turkey	29.4 km NE from Mardin to Midyat	1971	In this study
Ae. caudata L.	KU-5885	Turkey	210 km E of Adana 25 km W of Gaziantep	1968	In this study
Ae. caudata L.	KU-11420	Greece	Mt. Taygetos west of Anogia Peloponnesos	1981	In this study
Ae. caudata L.	KU-11422	Greece	44 km S from Kalamata to Areopoli Peloponnesos	1981	In this study
Ae. caudata L.	KU-12066	Greece	10.1 km E from Irakrion to Gournes Crete	1983	In this study
Ae. caudata L.	KU-12095	Greece	5 km W from Kokarion to Karlovasi Samos	1983	In this study
Ae. caudata L.	KU-12136	Greece	4.7 km N from Voutas to Kastelli Crete	1983	In this study
Ae. caudata L.	KU-12163a	Greece	12.1 km NE from Selia to Rethimnon Crete	1983	In this study
Ae. comosa ssp. *heldreichii*	KU-5814	Greece	Chania - Souda Crete Island	1960	In this study
Ae. comosa ssp. *heldreichii*	KU-11447	Greece	31 km E from Agios Petros to Astros Peloponnesos	1981	In this study
Ae. comosa ssp. *heldreichii*	KU-11469	Greece	Mt. Taygetos west of Anogia Peloponnesos	1981	In this study
Ae. uniaristata Vis.	KU-11479	Greece	11 km SW from Megalopoli to Kiparissa Peloponnesos	1981	In this study
Ae. uniaristata Vis.	PI 554418	Unknown	Former Soviet Union	1991	In this study
Ae. uniaristata Vis.	PI 554420	Turkey	İzmir	1991	In this study

Table S2 Quality control of RNA-seq reads of C, M, N-genome diploid wheat accessions

Species	Accession number	Read pairs	Filtered read pairs	Forward only surviving reads	Reverse only surviving reads	Dropped reads
Ae. caudata	KU-5482	4,878,238	3,007,539 (61.65%)	1,296,886 (26.59%)	118,646 (2.43%)	45,5167 (9.33%)
	KU-5484	5,511,091	3,256,654 (59.09%)	1,072,465 (19.46%)	433,393 (7.86%)	748,579 (13.58%)
	KU-5885	5,365,936	3,226,284 (60.13%)	1,515,186 (28.24%)	119,700 (2.23%)	504,766 (9.41%)
	KU-11420	4,530,173	3,115,820 (68.78%)	883,880 (19.51%)	96,859 (2.14%)	433,614 (9.57%)
	KU-11422	5,435,011	4,276,369 (78.68%)	553,693 (10.19%)	120,142 (2.21%)	484,807 (8.92%)
	KU-12066	5,653,238	3,647,304 (64.52%)	749,482 (13.26%)	602,123 (10.65%)	654,329 (11.57%)
	KU-12095	5,060,173	2,955,794 (58.41%)	1,527,598 (30.19%)	89,223 (1.76%)	487,558 (9.64%)
	KU-12136	5,503,810	3,643,842 (66.21%)	645,124 (11.72%)	612,048 (11.12%)	602,796 (10.95%)
	KU-12163a	4,657,471	3,617,448 (77.67%)	497,762 (10.69%)	108,768 (2.34%)	433,493 (9.31%)
Ae. comosa	KU-5814	5,195,198	3,102,697 (59.72%)	956,547 (18.41%)	436,536 (8.40%)	699,418 (13.46%)
	KU-11447	4,864,165	3,815,863 (78.45%)	572,147 (11.76%)	94,129 (1.94%)	382,026 (7.85%)
	KU-11469	6,277,837	5,040,664 (80.29%)	639,609 (10.19%)	169,835 (2.71%)	427,729 (6.81%)
Ae. uniaristrata	KU-11479	6,296,846	4,975,513 (79.02%)	623,202 (9.90%)	141,941 (2.25%)	556,190 (8.83%)
	PI554418	4,707,424	3,685,423 (78.29%)	541,480 (11.50%)	97,519 (2.07%)	383,002 (8.14%)
	PI554420	4,734,106	3,738,919 (78.98%)	531,601 (11.23%)	95,916 (2.03%)	367,670 (7.77%)

Table S3 Alignment rate and SNPs and indels for C, M, and N genome species

Species	Strain name	Nuclear genome			Chloroplast genome		
		Alignment rate	SNPs	Indels	Alignment rate	SNPs	Indels
Ae. caudata	KU-5482	81.93%	66,834	953	17.06%	154	20
	KU-5484	95.25%	20,739	223	11.13%	106	13
	KU-5885	85.68%	69,331	998	8.12%	120	15
	KU-11420	96.68%	13,401	177	17.52%	141	15
	KU-11422	74.77%	133,335	1,646	1.97%	89	10
	KU-12066	71.60%	109,410	1,543	2.44%	97	11
	KU-12095	94.61%	26,706	308	9.82%	160	16
	KU-12136	79.56%	77,056	1,203	11.28%	137	10
	KU-12163a	73.09%	135,902	1,602	1.67%	63	7
Ae. comosa	KU-5814	94.18%	23,551	292	11.51%	144	19
	KU-11447	97.24%	14,880	220	21.80%	184	21
	KU-11469	85.92%	86,171	1,528	33.08%	205	18
Ae. uniaristata	KU-11479	66.86%	184,593	2,273	0.52%	31	9
	PI554418	95.19%	23,646	303	15.33%	170	20
	PI554420	96.18%	20,901	278	13.76%	151	14

Table S4 Polymorphic sites in *Ae. caudata* (CC genome), *Ae. comosa* (MM genome) and *Ae. uniaristata* (NN genome)

Species	Chr.1	Chr.2	Chr.3	Chr.4	Chr.5	Chr.6	Chr.7	Total
Ae. comosa	8,359	9,398	9,028	8,044	10,586	7,720	8,569	61,704
Ae. uniaristata	14,211	16,505	16,278	13,829	17,992	13,090	14,747	106,652
Ae. caudata	11,790	13,136	12,403	10,733	13,572	10,492	10,892	83,018

Number of accessions of *Ae. comosa*, *Ae. uniaristata*, and *Ae. caudata* is three, three, nine, respectively.

Table S5 Fixed SNPs between *Ae. caudata* (CC genome), *Ae. comosa* (MM genome) and *Ae. uniaristata* (NN genome)

Comparisons	Chr.1	Chr.2	Chr.3	Chr.4	Chr.5	Chr.6	Chr.7	Total
Ae. comosa vs. *Ae. uniaristata*	1,729	2,025	1,945	1,828	2,088	1,736	2,249	13,600
Ae. comosa vs. *Ae. caudata*	10,881	12,252	11,291	10,247	12,903	10,216	10,181	77,971
Ae. uniaristata vs. *Ae. caudata*	12,997	14,679	13,649	12,378	14,957	11,731	11,789	92,180

Table S6 List of CAPS markers to distinguish M and N genomes

Name	Chr.	Sequence(5’ to 3’)	Restriction enzyme	Annealing temp. (℃)
N010_F	Chr.3	TTCATTTCTCATCCCCGCAG	*Taq*αI	58
N010_R		ACCAAGCAGTTCACCTGAAG		
M008_F	Chr.4	TGTGAGCCCAGTTAGTGGTA	*Dde* I	54
M008_R		TGTGTGCACATGTGAATGGA		
MN002_F	Chr.5	GGACAGGTACCTCTTTCCCT	*Vpa*k11B I	54
MN002_R		TGTCACCGTAGTGTACCTGT		
N002_F	Chr.6	GCTGCTCTGTTTCTTTGCTG	*Sal* I	54
N002_R		CAGTCCTCTGTTAGCTGCAG		

Table S7 Plant materials used for phylogenetic and polymorphic analyses

Species	Strain name	Country	Locality	Year	RNA-seq read data
Ae. tauschii	KU-2025	Afganistan	22 km N of Doski (Kabul - Pulikhumri)	1956	DRA004604 (Nishijima et al. 2016)
Ae. tauschii	AT76	China	Unknown	Unknown	DRA004604 (Nishijima et al. 2016)
Ae. tauschii	KU-2003	Pakistan	Suburbs of Quetta	1956	DRA004604 (Nishijima et al. 2016)
Ae. tauschii	KU-2075	Iran	15 km E of Behshahr (Behshahr - Gorgan)	1956	DRA004604 (Nishijima et al. 2016)
Ae. tauschii	KU-2078	Iran	E of Aliabad (Gorgan - Khoshyailagh)	1956	DRA004604 (Nishijima et al. 2016)
Ae. tauschii	KU-2087	Iran	15 km NE of Sari (Sari - Behshahr)	1956	DRA004604 (Nishijima et al. 2016)
Ae. tauschii	KU-2093	Iran	51 km W of Babulsar (Babulsar - Chalus)	1956	DRA004604 (Nishijima et al. 2016)
Ae. tauschii	KU-2124	Iran	Techalousse (near Chalus)	1956	DRA004604 (Nishijima et al. 2016)
Ae. tauschii	KU-2627	Afganistan	5 km S from Jurm Badakhshan	1979	DRA004604 (Nishijima et al. 2016)
Ae. tauschii	PI 499262	China	Xinjiang Uygur Zizhiqu	1985	DRA004604 (Nishijima et al. 2016)
Ae. umbellulata Zhuk.	KU-4017	Iraq	18.8 km NNE from Sulaymaniyah to Chuarta	1971	DRA006404 (Okada et al. 2018)

Table S7 (continued)

Species	Strain name	Country	Locality	Year	RNA-seq read data
Ae. umbellulata Zhuk.	KU-4026	Iraq	25.9 km S from Kirkuk to Baghdad	1971	DRA006404 (Okada et al. 2018)
Ae. umbellulata Zhuk.	KU-4035	Iraq	5.5 km ENE from Koi Sanjak to Ranya	1971	DRA006404 (Okada et al. 2018)
Ae. umbellulata Zhuk.	KU-4043	Iraq	SSW of Rowanduz	1971	DRA006404 (Okada et al. 2018)
Ae. umbellulata Zhuk.	KU-4052	Iraq	4.4 km NW from Amadiyah Mazorka Gorge	1971	DRA006404 (Okada et al. 2018)
Ae. umbellulata Zhuk.	KU-4103	Turkey	North of Van	1971	DRA006404 (Okada et al. 2018)
Ae. umbellulata Zhuk.	KU-5934	Turkey	Suburbs of Kayseri	1960	DRA006404 (Okada et al. 2018)
Ae. umbellulata Zhuk.	KU-5954	Turkey	Suburbs of Kutahia	1980	DRA006404 (Okada et al. 2018)
Ae. umbellulata Zhuk.	KU-12180	Greece	5.1 km W from Platania to Laerma Rhodes	1983	DRA006404 (Okada et al. 2018)
Ae. umbellulata Zhuk.	KU-12198	Greece	5.4 km E from Mithymna to Madamados Lesbos	1983	DRA006404 (Okada et al. 2018)
Ae. umbellulata Zhuk.	KU-8-5	Syria	6 km W of Qatana (Damascus - Mt. Hermon)	1960	DRA006404 (Okada et al. 2018)
Ae. umbellulata Zhuk.	KU-8-7	Turkey	Suburbs of Burdur (D)	1960	DRA006404 (Okada et al. 2018)

Table S7 (continued)

Species	Strain name	Country	Locality	Year	RNA-seq read data
T. monococcum ssp. *aegilopides*	KU-101-3	Iran	Collection of Agr. Exp. Station Tehran	1956	DRA007574 (Michikawa et al. 2019)
T. monococcum ssp. *aegilopides*	KU-3620	Turkey	51 km S of Ankara (Ankara - Adana)	1960	DRA007574 (Michikawa et al. 2019)
T. monococcum ssp. *aegilopides*	KU-3646	Turkey	69 km NE of Urfa	1968	DRA007574 (Michikawa et al. 2019)
T. monococcum ssp. *aegilopides*	KU-8111	Iraq	5.5 km ENN from Koi Sanjaq to Ranya	1971	DRA007574 (Michikawa et al. 2019)
T. monococcum ssp. *aegilopides*	KU-8120	Iraq	10.6 km ENE from Koi Sanjaq to Ranya	1971	DRA007574 (Michikawa et al. 2019)
T. monococcum ssp. *aegilopides*	KU-8267	Iraq	19.6 km S from Zakho to Mosul	1971	DRA007574 (Michikawa et al. 2019)
T. monococcum ssp. *aegilopides*	KU-8276	Iraq	25.6 km NNW from Mardin to Diyarbakir	1971	DRA007574 (Michikawa et al. 2019)
T. monococcum ssp. *aegilopides*	KU-8287	Turkey	38.6 km E from Diyarbakir to Silvan	1971	DRA007574 (Michikawa et al. 2019)
T. monococcum ssp. *aegilopides*	KU-10725	Turkey	42 km N of Ankara (Ankara - Bolu)	1977	DRA007574 (Michikawa et al. 2019)
T. monococcum ssp. *aegilopides*	PI 427634	Turkey	20.2 km E of Siverek	1978	DRA007574 (Michikawa et al. 2019)
T. urartu Thum.	KU-199-5	Turkey	Unknown	1977	DRA007574 (Michikawa et al. 2019)

Table S7 (continued)

Species	Strain name	Country	Locality	Year	RNA-seq read data
T. urartu Thum.	KU-199-11	Turkey	Kiziltep	1978	DRA007574 (Michikawa et al. 2019)
T. urartu Thum.	KU-199-16	Lebanon	Baal Bek	1978	DRA007574 (Michikawa et al. 2019)
T. monococcum ssp. *monococcum*	DV92	Italy	Unknown	Unknown	DRA007574 (Michikawa et al. 2019)
T. monococcum ssp. *monococcum*	KT-003-001	Unknown	Unknown	Unknown	DRA007574 (Michikawa et al. 2019)
Ae. speltoides ssp. *speltoides*	KU-2208A	Turkey	145 km W of Mardin (Urfa - Mardin)	1977	DRA007097 (Miki et al. 2019)
Ae. speltoides ssp. *speltoides*	KU-14601	Israel	Qishon river bank E of the bridge Haifa	1993	DRA007097 (Miki et al. 2019)
Ae. speltoides ssp. *speltoides*	KU-14605	Israel	Ramat haNadiv in Zikhron Ya'aqov	1993	DRA007097 (Miki et al. 2019)
Ae. speltoides ssp. *ligustica*	KU-2236	Turkey	96 km N of Maras (Kayseri - Maras)	1977	DRA007097 (Miki et al. 2019)
Ae. speltoides ssp. *ligustica*	KU-7716	Iraq	13.2 km S from Sulaymaniyah to Qara Dagh	1971	DRA007097 (Miki et al. 2019)
Ae. speltoides ssp. *ligustica*	KU-7848	Iraq	39.0 km NNW from Mosul to Amadiyah	1971	DRA007097 (Miki et al. 2019)

Table S7 (continued)

Species	Strain name	Country	Locality	Year	RNA-seq read data
Ae. speltoides ssp. *speltoides*	KU-12963a	Syria	Oppisite military airfield on the road Aleppo - Afrin Aleppo (Site No. 7551)	1985	DRA007097 (Miki et al. 2019)
Ae. bicornis (Forsk.) Jaub.et Sp.	KU-5784	Egypt	21 km W of Alexandria (A)(Alexandria - Cairo)	1960	DRA007097 (Miki et al. 2019)
Ae. bicornis (Forsk.) Jaub.et Sp.	KU-14613	Israel	2 km E of Kerem Shalom near km10 along Hwy 232	1993	DRA007097 (Miki et al. 2019)
Ae. longissima Schweinf. et Muschl.	KU-5752	Jordan	16 km E of Dead Sea (Dead Sea - Amman)	1960	DRA007097 (Miki et al. 2019)
Ae. longissima Schweinf. et Muschl.	KU-14624	Israel	Beit Hananya N of Zikhrom Ya'aqov at the Bus Stop along Hwy 4	1993	DRA007097 (Miki et al. 2019)
Ae. longissima Schweinf. et Muschl.	KU-14635	Israel	NE of Ammi'oz S of Gevulot Junction of Hwy 222 and Hwy 232 (ca 12km NW of Ze'elim)	1993	DRA007097 (Miki et al. 2019)
Ae. searsii Feld. et Kis.	KU-5755	Syria	Suburbs of Ramtha (Amman - Damascus)	1960	DRA007097 (Miki et al. 2019)
Ae. searsii Feld. et Kis.	KU-6142	Jordan	27 km before Jarash coming from Ramtha (border) Irbid (Site No. 7561)	1983	DRA007097 (Miki et al. 2019)

Table S7 (continued)

Species	Strain name	Country	Locality	Year	RNA-seq read data
Ae. searsii Feld. et Kis.	KU-6143	Jordan	Road from Mafraq to Jarash 13 km after Mafraq (before Rihab) Irbid (Site No. 7566)	1983	DRA007097 (Miki et al. 2019)
Ae. searsii Feld. et Kis.	KU-14651	Israel	Gitit rocky slope N of Hwy 508 (alt 320 m ca 9km W of Massu'a)	1993	DRA007097 (Miki et al. 2019)
Ae. sharonensis Eig	KU-14661	Israel	Ein Hamifratz south of Akko Along Hwy 4	1993	DRA007097 (Miki et al. 2019)
Ae. sharonensis Eig	KU-14663	Egypt	Beach in Dor haBonim Nature Reserve near haBonim	1993	DRA007097 (Miki et al. 2019)
Ae. sharonensis Eig	KU-14668	Israel	Wingate S of Netanya along Hwy 2	1993	DRA007097 (Miki et al. 2019)

Table S8 Alignment rate and SNPs and indels for diploid *Triticum* and *Aegilops* species used for phylogenetic and polymorphic analyses

Species	Strain name	Nuclear genome			Chloroplast genome		
		Alignment rate	SNPs	Indels	Alignment rate	SNPs	Indels
Ae. tauschii	KU-2003	70.51%	69,939	2,240	0.46%	9	2
(DD genome)	KU-2025	82.83%	25,478	725	17.84%	24	11
	KU-2075	93.48%	31,789	1,255	0.31%	10	3
	KU-2078	93.27%	28,072	1,079	0.52%	2	1
	KU-2087	91.35%	81,943	2,821	1.28%	10	3
	KU-2093	94.83%	27,615	1,058	0.35%	12	5
	KU-2124	94.66%	19,675	781	0.47%	14	5
	KU-2627	92.66%	79,845	2,636	0.45%	9	3
	PI499262	94.75%	38,524	1,221	3.10%	9	7
Ae. umbellulata	KU-4017	82.83%	85,137	895	15.11%	148	14
(UU genome)	KU-4026	95.83%	23,204	252	32.40%	149	12
	KU-4035	70.78%	145,479	1,961	1.98%	79	6
	KU-4043	78.29%	89,085	1,179	12.52%	119	12
	KU-4052	73.70%	96,143	1,177	4.63%	81	5
	KU-4103	91.98%	34,722	420	30.61%	160	14
	KU-5934	70.63%	140,081	1,877	1.48%	73	7
	KU-5954	74.91%	105,757	1,152	6.38%	96	10
	KU-12180	72.92%	101,498	1,235	5.25%	87	15
	KU-12198	72.97%	110,432	1,235	4.58%	83	6
	KU-8-5	70.14%	116,689	1,453	1.25%	58	6
	KU-8-7	70.51%	109,438	1,417	2.40%	63	9
T. monococcum ssp. *aegilopides*	KU-101-3	76.28%	103,704	975	12.24%	107	6
(A^mA^m genome)	KU-3620	73.70%	107,476	1,225	14.18%	131	10
	KU-3646	68.89%	134,173	1,490	7.54%	96	6
	KU-8111	78.77%	94,761	750	14.58%	87	5
.	KU-8120	78.62%	107,268	855	16.12%	125	7
	KU-8267	74.07%	109,568	861	9.74%	74	5
	KU-8276	75.67%	105,754	896	12.08%	82	7
	KU-8287	66.65%	164,738	1,578	3.19%	71	7
	KU-10725	81.58%	65,723	616	18.63%	143	10
	PI427634	94.16%	29,369	297	35.23%	164	8

Table S8 (continued)

Species	Strain name	Nuclear genome			Chloroplast genome		
		Alignment rate	SNPs	Indels	Alignment rate	SNPs	Indels
T. monococcum ssp. *monococcum*	DV92	84.02%	66,419	661	10.85%	113	8
(A^mA^m genome)	KT-003-001	94.16%	88,634	1,069	11.45%	91	5
T. urartu	KU-199-5	68.50%	112,635	1,488	8.00%	66	7
(AA genome)	KU-199-11	65.36%	150,654	1,574	2.25%	57	4
	KU-199-16	77.46%	100,278	847	11.07%	101	9
Ae. speltoides ssp. *speltoides*	KU-2208A	66.21%	84,479	1,092	9.29%	91	9
(SS genome)	KU-14601	65.72%	134,850	1,571	2.60%	141	13
	KU-14605	66.03%	121,099	1,337	4.29%	140	11
	KU-12963a	58.79%	124,602	1,525	4.07%	46	3
Ae. speltoides ssp. *ligustica*	KU-2236	60.29%	145,804	1,388	1.45%	47	7
(SS genome)	KU-7716	69.16%	91,616	1,096	11.04%	85	8
	KU-7848	66.66%	139,476	1,403	3.89%	129	19
Ae. bicornis	KU-5784	83.37%	76,097	900	26.36%	129	13
(S^bS^b genome)	KU-14613	92.91%	35,001	400	44.42%	128	15
Ae. longissima	KU-5752	80.90%	73,347	1,149	25.30%	122	15
(S^lS^l genome)	KU-14624	76.23%	104,479	1,298	3.59%	99	10
	KU-14635	70.91%	97,606	1,532	6.49%	95	14
Ae. searsii	KU-5755	90.70%	49,689	605	39.25%	147	22
(S^sS^s genome)	KU-6142	79.60%	126,493	1,335	16.25%	115	12
	KU-6143	74.94%	97,005	1,177	7.56%	113	12
	KU-14651	72.70%	126,493	1,621	6.59%	121	13
Ae. sharonensis	KU-14661	70.37%	95,530	1,798	5.02%	86	11
(S^lS^l genome)	KU-14663	73.64%	115,984	1,722	5.74%	98	13
	KU-14668	72.66%	90,749	1,331	8.97%	99	16
Hordeum vulgare (HH genome)	Harunanijo	28.14%	173,330	524	0.61%	220	11

Table S9 Sets of non-redundant SNPs in diploid *Triticum* and *Aegilops* species estimated using RNA-seq

Set	Chr.1	Chr.2	Chr.3	Chr.4	Chr.5	Chr.6	Chr.7	Total	Chloroplast
Without *H. vulgare*	15,053	16,781	15,688	15,126	18,421	15,064	13,847	109,980	166
With *H. vulgare*	14,849	16,793	15,330	14,946	18,461	14,421	13,818	108,618	234

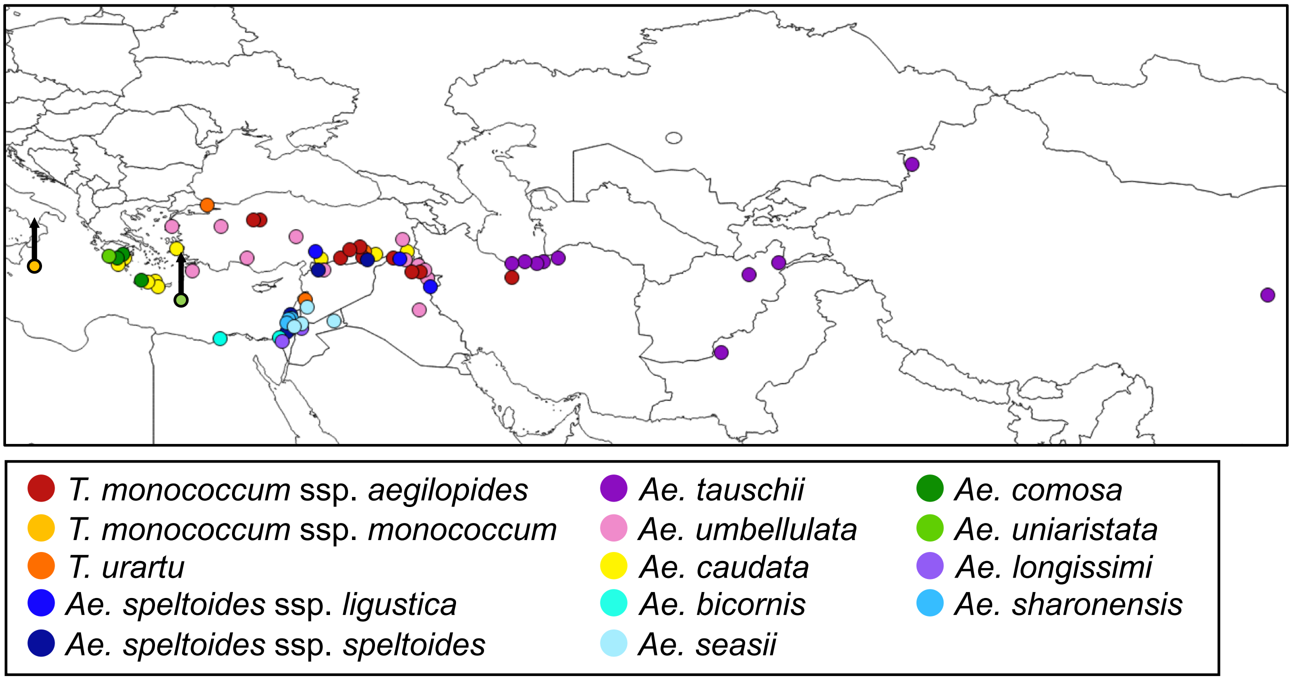

Fig. S1 Geographic distribution of diploid *Triticum* and *Aegilops* species that were analyzed in this study.

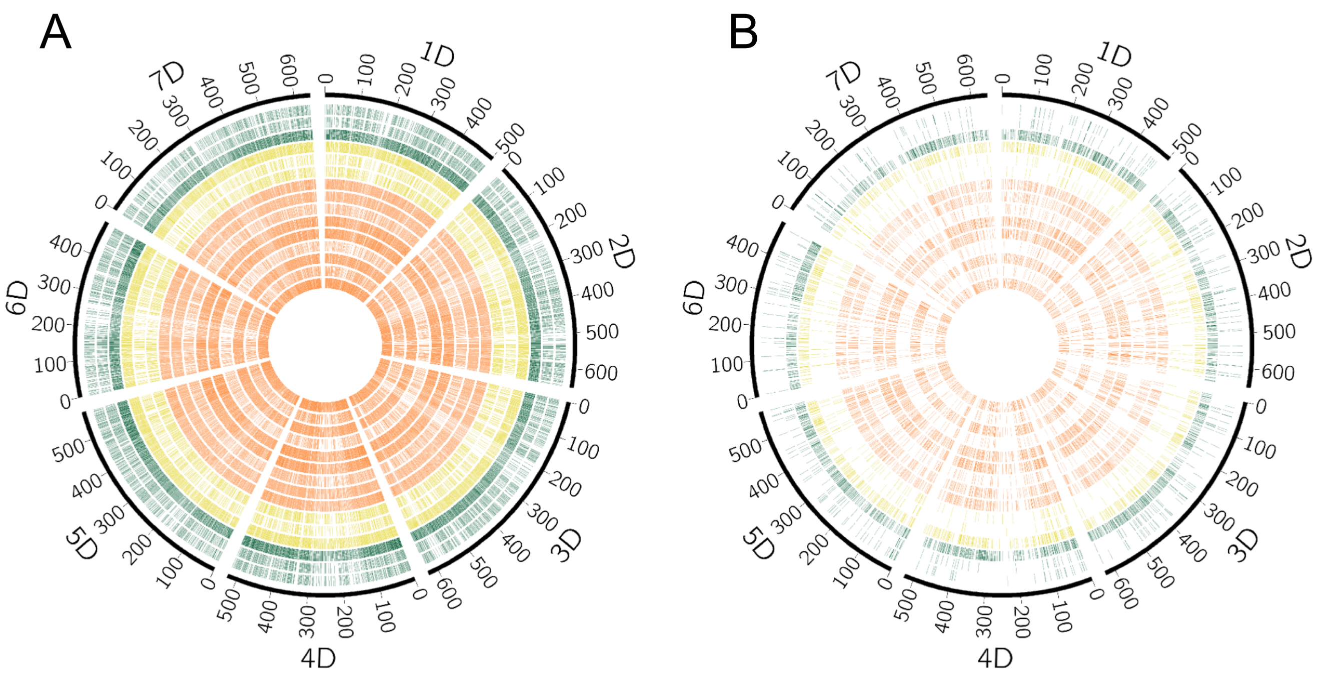

Fig. S2 Distribution of SNPs and indels of *Ae. caudata*, *Ae. comosa* and *Ae. uniaristata*. In the CIRCOS visualizations, green, yellow and orange indicates *Ae. uniaristata*, *Ae. comosa,* and *Ae. caudata*, respectively. SNPs (A) and indels (B) for *Ae. uniaristata* KU-11479, PI554418 and PI554420; *Ae. comosa* KU-5814, KU-11447, and KU-11469; and *Ae. caudata* KU-5482, KU-5484, KU-5885, KU-11420, KU-11422, KU-12066, KU-12095, KU-12136, and KU-12136a are shown from the outer to the inner circles.

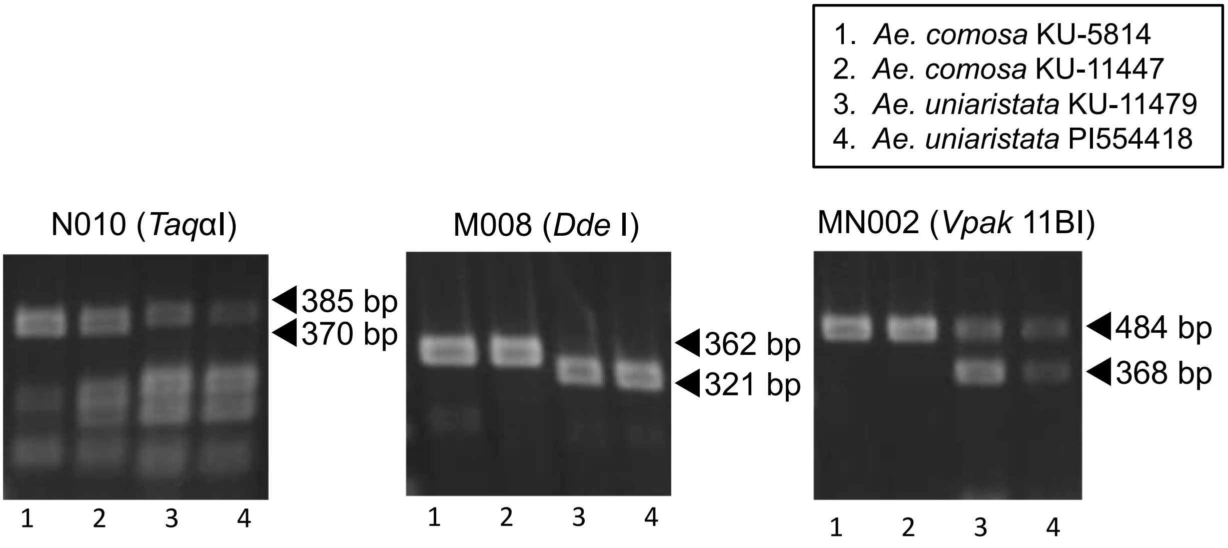

Fig. S3 CAPS markers that discriminate M or N genomes from A and B genomes.

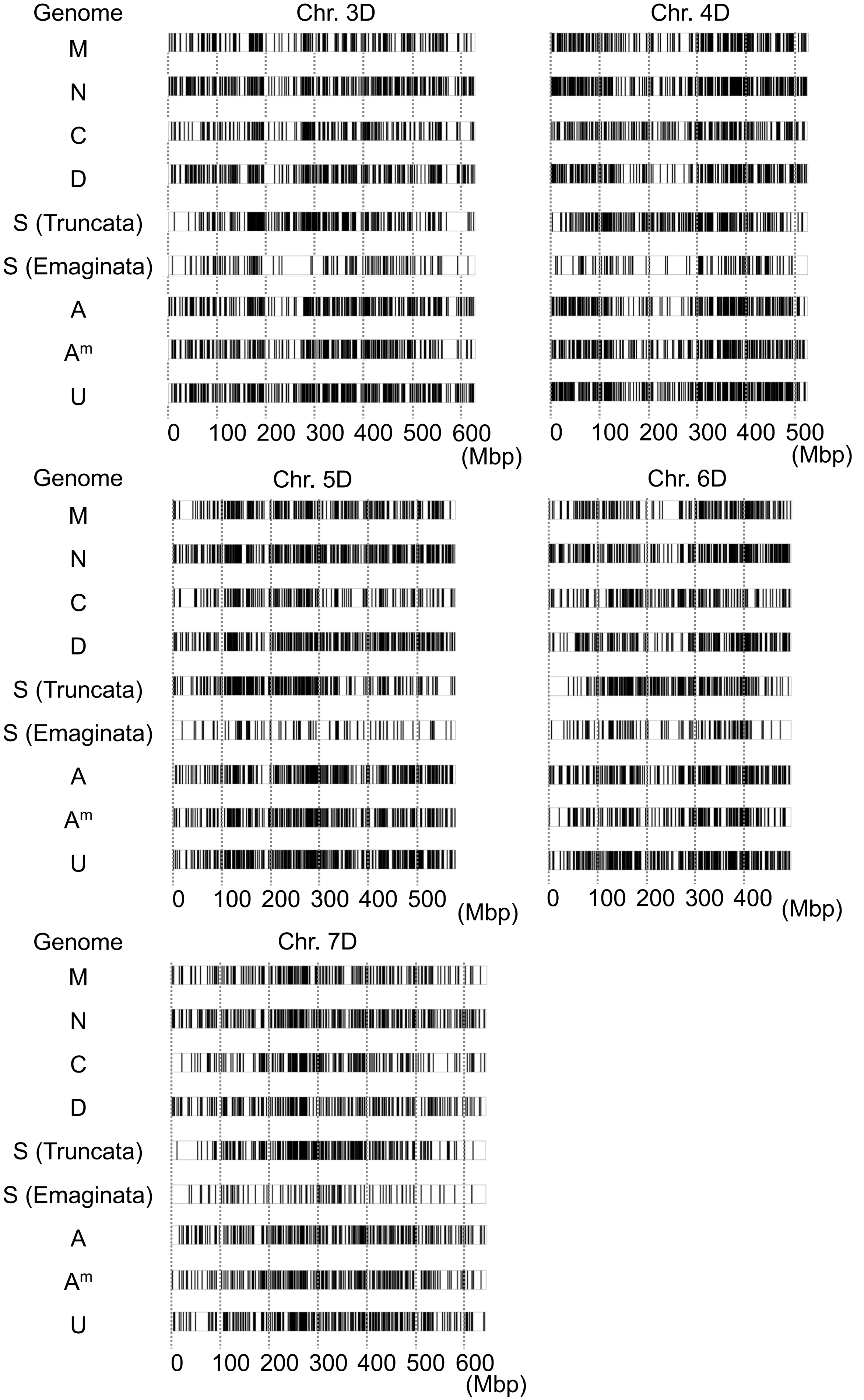

Fig. S4 Distribution of unique SNPs that discriminate other genomes over each chromosome. The unique SNPs for each genome were mapped to the chromosomes of *Ae. tauschii*. Black bars indicate SNP positions. The figure shows the distribution of the unique SNPs on the chromosomes 3D, 4D, 5D, 6D, and 7D.
